# Adenosine Concentration in Patients With Neurally Mediated Syncope

**DOI:** 10.3389/fcvm.2022.900023

**Published:** 2022-06-17

**Authors:** Antonella Groppelli, Michele Brignole, Mohamed Chefrour, Marguerite Gastaldi, Farid El Oufir, Jean Claude Deharo, Gianfranco Parati, Régis Guieu

**Affiliations:** ^1^Cardiology Unit, Faint & Fall Programme, Department of Cardiology, IRCCS Istituto Auxologico Italiano, San Luca Hospital, Milan, Italy; ^2^Laboratory of Biochemistry, Timone Hospital, Marseille, France; ^3^C2VN, Aix Marseille University, Marseille, France; ^4^Department of Cardiology, Timone Hospital, Marseille, France; ^5^Department of Medicine and Surgery, University of Milano Bicocca, Milan, Italy

**Keywords:** adenosine, syncope, neurally-mediated syncope, methodology, adenosine receptors

## Abstract

**Background:**

Either high or low values of adenosine blood level (ABL) can differentiate some forms of neurally mediated syncope (NMS). A rapid method of measurement has recently been developed. The aim of the present study was: (1) to compare ABLs in an unselected population of consecutive patients referred for evaluation of suspected NMS syncope and in healthy controls; and (2) to assess the relative prevalence of low and high adenosine forms among an unselected syncope population.

**Method:**

Whole blood was collected after finger puncture, blood being deposit on a blot paper and adenosine concentration was measured by liquid chromatography/mass spectrometry (LC-MS/MS).

**Results:**

Among 89 control subjects, the median ABL value was 0.54 μM (IQR, 0.46–0.65). The lowest 5% and the upper 95% percentile were 0.40 and 0.80 μM, respectively. Compared with healthy subjects, the 146 patients with syncope showed, on average, a higher median ABL value [0.63 (IQR 0.45–0.73, *p* = 0.04)] and a larger distribution of values. Low ABL values below the 5th percentile were observed in 28 (19%) patients, and, in five controls, *p* = 0.003 and high ABL values were observed in 26 (18%) patients and five controls, *p* = 0.009.

**Conclusions:**

ABL is different in patients with suspected NMS than in healthy subjects. Patients with low and high adenosine values account for 19% and 18% of the general population. Thus, low and high ABL limits, as defined in this study, may help to define the purinergic profile of unselected subjects with a clinical diagnosis of suspected NMS.

## Introduction

Either central or peripheral baroreceptor reflex abnormalities and/or alterations in neurohumoral mechanisms could play a pivotal role in the genesis of extrinsic (functional) and neurally mediated syncope (NMS) (1). Among the several biochemical mediators that are advocated to play a role, adenosine has recently been investigated in typical vasovagal syncope and in syncope without prodromes in subjects with a normal heart. Either high- or low-adenosine values have been reported to be able to differentiate some forms of syncope, but adenosine does not seem to be involved in other forms. Schematically, high-adenosine and low-adenosine forms have been identified (2). When baseline adenosine is low, the effect of adenosine on the AV node and the sino-atrial node is mainly due to the stimulation of high-affinity A_1_ receptors, (A_1_R) which are much more numerous in the AV node than in the sino-atrial node. The effects of adenosine on A1 R leads to bradycardia, sinus arrest or atrio-ventricular block (3, 4). Conversely, the high adenosine values are compatible with the activation of low-affinity A_2A_ receptors (A_2A_R) that are in the vessels and which cause vasodilation (2).

The prevalence of patients with low and high adenosine syncope is uncertain because, until now, adenosine has been studied in small, selected populations. A barrier to the widespread evaluation of adenosine in clinical practice has been the difficulty of obtaining rapid reliable measures. Indeed, owing to its short half-life in the blood, adenosine is not easy to sample and measure. At the time of venepuncture, a stop solution – not commercially available - must be utilized to inhibit the degradation of adenosine in body fluid. After plasma deproteinization, adenosine concentration is evaluated by means of high-performance liquid chromatography. A rapid method (“blot spot”) has recently been developed and assessed in a small population (5). It consists of measuring adenosine concentration in whole blood instead of plasma, using fixed potential amperometry. This method is rapid, well-accepted by the patient, not expensive, and easily replicable.

The aim of the present study was: (1) to compare ABLs in an unselected population of subjects with a history of suspected NMS and in healthy controls; and (2) to assess the relative prevalence of low and high adenosine forms among this population.

## Methods

We assessed the rapid method of adenosine dosage in whole blood using Liquid Chromatography, Mass Spectrometry (LC-MS/MS) (5) in unselected consecutive patients with a history of suspected NMS and in healthy volunteers. The blood samples were shipped to the Laboratory of Biochemistry of Timone Hospital for analysis.

The Syncope group was formed by patients aged >14 years referred to the syncope clinic of Istituto Auxologico Italiano, Milan, Italy because affected by one or more episodes of unexplained syncope to confirm or reject the clinical suspicion of NMS after exclusion of competing diagnoses. In particular, according to the diagnostic criteria of ESC guidelines (6), the patients with likely cardiac syncope and those with a transient loss of consciousness of likely non-syncopal origin were excluded. The patients underwent head-up tilt test (HUTT) according to the Italian protocol (7).

### Blood Samples Collection

The day of blood sampling, the participants were allowed to take their usual drug therapies and had no dietary restrictions. Whole blood was collected using finger puncture, while the patient was sitting in a quiet environment, followed by deposit of a drop of blood (20 μL) on a blotting paper (Whatman 903 protein saver cards™) and dried overnight at room temperature to obtain dried blood spot. Then, the blot spots were stored at room temperature and shipped to the Laboratory of Biochemistry of Timone Hospital for analysis.

### Blood Samples Extraction

The method has been previously described (5). Briefly, six millimeters of dried blood spot were cut out, followed by extraction with mixture, consisting of methanol and internal standards mixed for 90 min at 45°C. After extraction, aliquots (350 μL) were transferred into a new 2-ml safe-lock tube and evaporated to dryness at 60°C under nitrogen; 150 μl of 0.1% formic acid in water was added and quickly vortexed before transferring into an HPLC auto sampler vial.

### Adenosine Dosage

After the extraction, adenosine concentration was measured by LC-MS/MS. Samples were analyzed using a Shimadzu UFLC XR system (Shimadzu, Marne la Vallee, France). The LC system was interfaced with an ABSciex 4,500 triple quadrupole mass spectrometer (Les Ulis, France), operating with an electrospray ionization source (ESI) using nitrogen (purity: 99.99%). Ten microliters of the extracted sample were injected onto a 2.1-mm- × -100-mm, 3-μm Atlantis R T3 column, Waters (Guyancourt, France). The starting mobile phase consisted of 3% methanol and 97% acidified water (0.1% formic acid), with a flow of 0.7 ml/min for 3.5 min. Then, the gradient of methanol was increased to 30% for 3 min. The column was re-equilibrated for 2 min to starting conditions.

### Statistical Analysis

ABL values were reported as median and interquartile range [IQR] and compared between groups by means of Mann–Whitney non-parametric test. Categorical variables were shown as absolute and relative frequencies and compared between groups by means of the Fisher exact test or Chi-squared test for a trend as appropriate.

## Results

The study population consisted of 146 patients with a diagnosis of likely neurally mediated syncope; their mean age was 57 ± 20 years, 67 were males (46%). The clinical features of patients with syncope were consistent with those of an unselected general population: 108 had prodromes, suggesting an autonomic activation, 54 had recognizable triggers (orthostatic, emotional, situational or post-prandial, single or in combination); 55 were taking antihypertensive drugs. HUTT was positive in 101 (72%) patients (vasodepressor or mixed in 88 and cardioinhibitory in 13) and negative in 45 (28%) patients. The control group consisted of 89 healthy volunteers; their mean age was 51 ± 18 years, 46 were males (52%).

**Figure 1:**
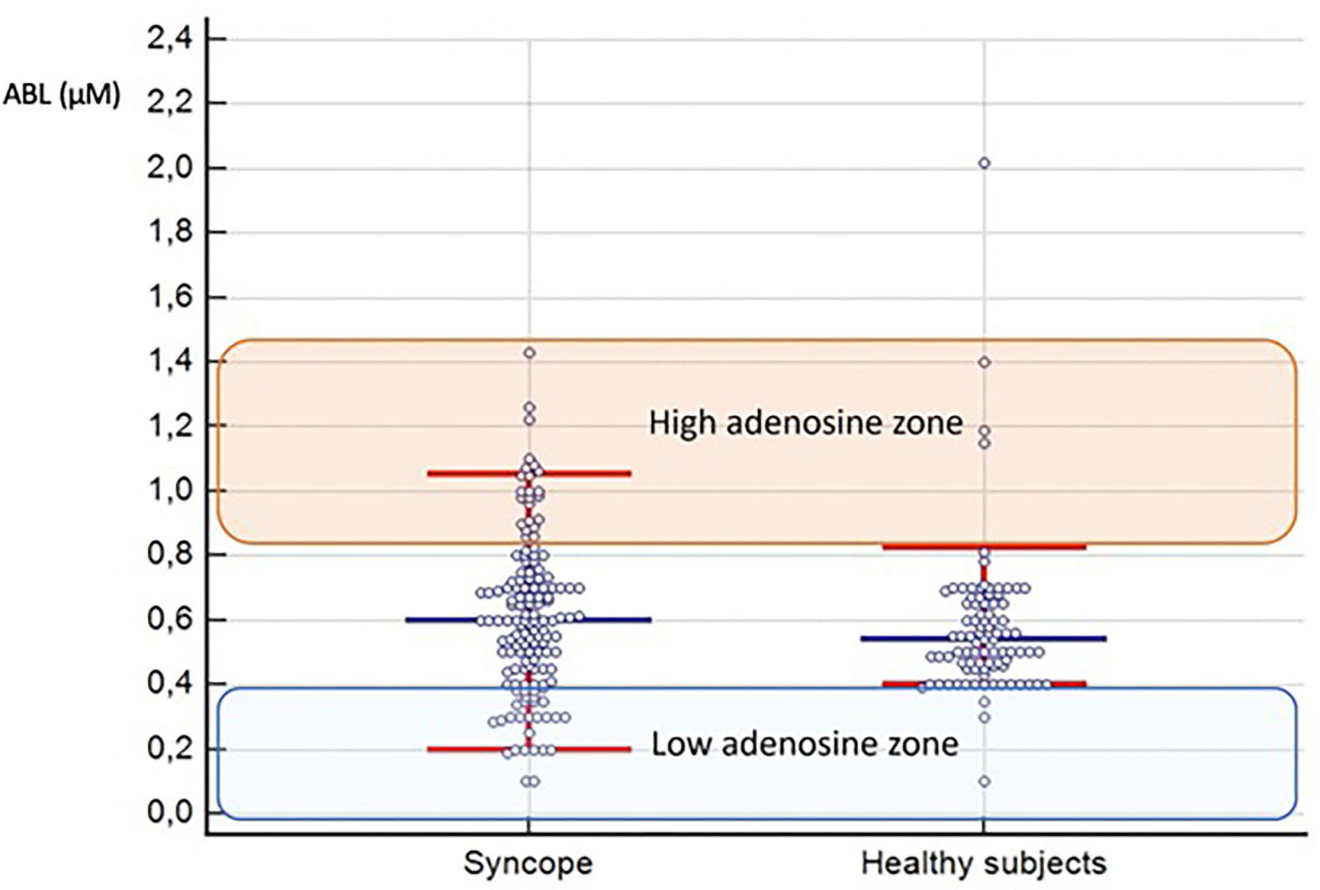
Distribution of patients with syncope and healthy controls based on their adenosine levels. The blue horizontal line shows the median; the red horizontal lines show the 5th and the 95th percentiles of the two groups. The low adenosine zone and the high adenosine zone, based on the normal ABL range in control subjects, are identified as well.

In controls, the average median ABL value was 0.54 μM (IQR, 0.46–0.65). The lowest 5th and the highest 95th percentiles were 0.40 μM and 0.80 μM, respectively; these values were, therefore, considered as the normal limit for low ABL and high ABL in patients with syncope. Table 1 shows the results of ABL dosage in both groups. Compared with healthy subjects, the patients with NMS showed, on average, a higher median ABL value [0.63 (IQR, 0.45–0.73, *p* = 0.04)] and a larger distribution of values (Figure 1). The low ABL zone included 28 (19%) patients and five controls, *p* = 0.003, and the high APL zone included 26 (18%) patients and five controls, *p* = 0.009.

**Table 1:** Comparison of adenosine values between healthy controls and patients with syncope by the blot spot method.

	**Healthy subjects (*n* = 89)**	**Syncope patients (*n* = 146)**	**Odds ratio**	***P*-value**
ABL, μM, median (IQR)	0.54 (0.46–0.65)	0.63 (0.45–0.73)		0.04
No. of pts with ABL <0.40 μM	5 (5.6%)	28 (19%)	4.0 (1.5–10.7	0.003
No. of pts with ABL >0.80 μM	5 (5.6%)	26 (18%)	3.6 (1.3–9.9)	0.009

**Figure 2:**
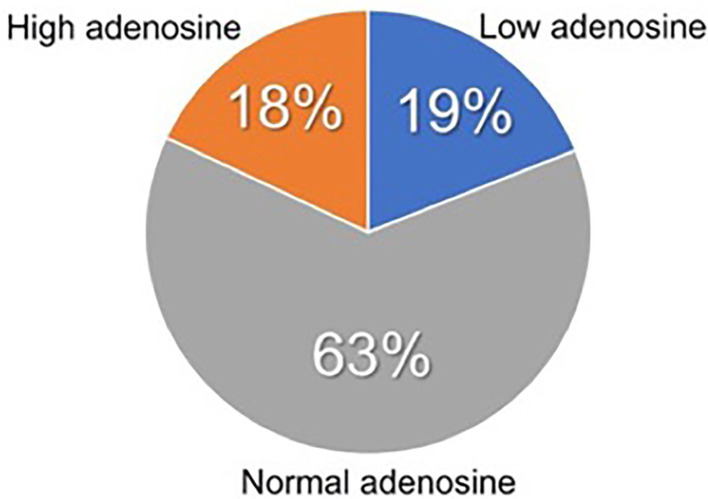
Prevalence of patients with low ABL syncope and with high ABL syncope among an unselected population of adult patients with NMS.

Thus, the phenotype of low adenosine syncope could be identified in 19% and that of high adenosine syncope in 18% of the whole syncope population (Figure 2). The clinical features of patients with low, normal, and high ABL are shown in Table 2. The clinical differences were modest, not reaching the level of significance.

**Table 2:** Clinical features according to adenosine values.

	**Low adenosine (*n* = 28)**	**Normal adenosine (*n* = 92)**	**High adenosine (*n* = 26)**	***P*-value**
Median ABL (IQR)	0.30 (0.20–0.35)	0.60 (0.52–0.70)	0.98 (0.89–1.06)	
Mean age	54 ± 22	57 ± 19	60 ± 19	0.62
Males	17 (61%)	40 (43%)	10 (38%)	0.19
Antihypertensive drugs	8 (29%)	38 (41%)	9 (35%)	0.47
Atypical or short prodromes^*^	8 (29%)	24 (26%)	6 (23%)	0.89
Orthostatic trigger	3 (11%)	21 (23%)	3 (12%)	0.21
Emotional trigger	3 (11%)	8 (9%)	0 (0%)	0.26
Situational syncope	4 (14%)	5 (5%)	3 (12%)	0.25
Post-prandial syncope	2 (7%)	12 (13%)	0 (0%)	0.12
Tilt test rersults:				
-Positive (*n* = 101)	20 (71%)	70 (76%)	17 (65%)	0.53
-Negative (*n* = 45)	8 (29%)	22 (24%)	9 (35%)	

* *Patients without typical triggers and/or without typical signs of activation of autonomic system*.

## Discussion

The main findings of the study are that rest ABL, measured with the rapid “blot spot” method on whole blood, can distinguish a general population of patients with suspected NMS from healthy subjects. Among an unselected NMS population, two groups of patients with low and high outlier ABL values can be identified, which account for 19% and 18% of the population, respectively. These results are consistent with those of previous studies performed with the standard method of high-performance liquid chromatography in plasma with stop solution (3, 4, 8, 9). The normal range of ABL observed with the “blot spot” method was similar to that of 0.40 to 0.78 μM observed with the chromatographic method in the laboratory of Biochemistry of Timone Hospital in 120 healthy subjects (10).

In our previous studies (3, 4, 8, 9), we showed that several patients with syncope without prodromes and normal heart had value of adenosine plasma levels below the 5^th^ percentile of healthy subjects and that several patients with positive vasovagal response during tilt testing had values above the 95th percentile of healthy subjects. The result of the present study shows that such correlation is more difficult to be found in an unselected general population. In a recent trial (10), the efficacy of theophylline, an adenosine receptor antagonist, was similar in patients with low and high AP measured with the chromatographic method, suggesting a lack of correlation between adenosine and clinical effect. Thus, it seems that the identification of low-adenosine syncope and high-adenosine syncope is more effective when the dosage of adenosine is performed in well-selected population affected by typical severe forms of syncope without prodromes and normal heart (3, 10) or in patients with typical features of vasovagal syncope as was the case in the pivotal studies (3, 9).

In conclusion, in this study, we have validated a rapid method (“blot spot”) for rapid, easy-to-perform, and cheap measurement of adenosine in the whole blood. The low and high ABL limits, as defined in this study, may help to define the purinergic profile of patients affected by NMS and can be used in future studies. Because the expression level of adenosine receptor is also crucial to interpret the relationship between the adenosinergic system and NHS, further studies are necessary to explore notably the ratio A_1_ R or A_2A_R expression/adenosine level ratio.

### Limitation of the Study

Many subjects with NMS were on antihypertensive treatment, which can modify plasma adenosine values. In previous studies, antihypertensive therapy was stopped at the time of adenosine blood sampling (8, 9, 11, 12). Furthermore, we did not ask our patients to stop coffee or tea before the study, although caffeine modifies the concentration of plasma adenosine (13) and makes the receptors more sensitive to the action of adenosine (14, 15) and thus may influence the occurrence of syncope. We did not take this step because we wanted to study the basal values of adenosine in the daily life of patients without changing their lifestyle. Similarly, tilt test was not performed in fasting state that could have influenced its result.

## Data Availability Statement

The raw data supporting the conclusions of this article will be made available by the authors, without undue reservation.

## Ethics Statement

Ethical review and approval was not required for the study on human participants in accordance with the local legislation and institutional requirements. The patients/participants provided their written informed consent to participate in this study.

## Author Contributions

AG: conceptualization, investigation, data curation, methodology, writing–original draft, and writing-review and editing. MB: conceptualization, formal analysis, methodology, writing–original draft, and writing-review and editing. MC and MG: adenosine measurement. FE: data collection. JD and GP: investigation and writing–review and editing. RG: conceptualization, investigation, and writing–review and editing. All authors contributed to the article and approved the submitted version.

## Funding

This work was supported by Amidex Méditerranée Foundation and Aix Marseille University.

## Conflict of Interest

The authors declare that the research was conducted in the absence of any commercial or financial relationships that could be construed as a potential conflict of interest.

## Publisher's Note

All claims expressed in this article are solely those of the authors and do not necessarily represent those of their affiliated organizations, or those of the publisher, the editors and the reviewers. Any product that may be evaluated in this article, or claim that may be made by its manufacturer, is not guaranteed or endorsed by the publisher.
